# A Clinical and Economic Framework for Identifying Potentially Avoidable Infection-Related Costs Following Minimally Invasive Aesthetic Procedures

**DOI:** 10.7759/cureus.110520

**Published:** 2026-06-09

**Authors:** Alejandro Rodriguez Bobadilla

**Affiliations:** 1 Infectious Diseases, Universidad del Rosario, Bogotá, COL

**Keywords:** aesthetic medicine, biofilm, botulinum toxin, cosmetic infections, health care costs, infection prevention, minimally invasive aesthetic procedures, nontuberculous mycobacteria, patient safety

## Abstract

Minimally invasive aesthetic procedures have steadily increased because of their accessibility, rapid recovery, and perceived low risk. However, disruption of the skin barrier, implantation of materials, manipulation of injectable products, and variability in biosafety practices may favor uncommon but clinically relevant infectious complications. This technical report proposes a clinical-economic framework to identify the clinical burden, health care resource use, and potentially avoidable costs associated with infections following procedures such as dermal fillers, botulinum toxin, mesotherapy, thread lifts, and other facial or body aesthetic interventions.

The framework integrates clinical pathways in which infections may present as cellulitis, abscesses, persistent inflammatory nodules, granulomas, biofilm-associated complications, or delayed infections caused by rapidly growing nontuberculous mycobacteria, particularly *Mycobacterium abscessus, Mycobacterium chelonae, *and* Mycobacterium fortuitum*. These complications may lead to diagnostic delay, repeated empirical antibiotic use, special cultures, biopsy, imaging, drainage, debridement, material removal, combined antimicrobial therapy, and prolonged specialist follow-up.

Their impact extends beyond the initial infectious event and may include aesthetic sequelae, psychological morbidity, loss of continuity of care, and substantial health care resource consumption. Potentially avoidable costs are concentrated in preventable process failures, including inadequate patient selection, deficient antisepsis, improper supply handling, products without traceability, absence of infection prevention protocols, low suspicion of mycobacterial infection, and delayed specialist referral. Infection prevention, product traceability, patient education, timely microbiological diagnosis, and standardized clinical pathways are essential components for reducing the clinical and economic burden of these complications.

## Introduction

Minimally invasive aesthetic procedures have steadily increased because of their accessibility, short recovery times, perceived natural results, and broad availability in outpatient settings. These procedures include dermal fillers, botulinum toxin, mesotherapy, thread lifts, biostimulatory treatments, microneedling, and combined facial or body rejuvenation techniques. Although they are often considered low risk when performed under appropriate safety standards, they share a critical infectious disease concern: disruption of the skin barrier, manipulation of injectable products or materials, and, in some cases, temporary or persistent implantation of substances into soft tissues [1,2].

Infectious complications after these procedures are uncommon relative to the overall volume of interventions performed; however, they may have considerable clinical relevance. Acute bacterial infections usually present as cellulitis, abscesses, folliculitis, local suppuration, or soft tissue infection. In contrast, subacute or delayed presentations pose greater diagnostic challenges, particularly when they appear as persistent nodules, granulomas, recurrent inflammatory lesions, or indolent abscesses. In this context, rapidly growing nontuberculous mycobacteria, including *Mycobacterium abscessus*, *Mycobacterium chelonae*, and *Mycobacterium fortuitum*, have been described as relevant pathogens after cosmetic procedures, injections, mesotherapy, dermal fillers, botulinum toxin, and procedures associated with medical tourism [3-6].

The impact of these infections extends beyond the initial clinical episode. Delayed presentation, low microbiological suspicion, insufficient response to empirical antibiotics directed against common skin flora, and the need for specialized diagnostic techniques may delay appropriate treatment. In many cases, management requires bacterial and mycobacterial cultures, antimicrobial susceptibility testing, biopsy, histopathology, imaging studies, drainage, debridement, material removal, combined antimicrobial therapy, and prolonged follow-up by dermatology, infectious diseases, plastic surgery, or aesthetic medicine specialists [6,7]. These clinical trajectories may generate cumulative health care resource use and may be associated with scarring, deformity, hyperpigmentation, loss of the expected aesthetic result, psychological distress, temporary disability, and deterioration of patient trust in the health care system [3,7].

Several modifiable process failures may amplify this burden, including inadequate patient selection, deficient antisepsis, inappropriate supply handling, incomplete sterilization control, absence of standardized infection-prevention protocols, limited post-procedure education, and late specialist referral [8-10]. Incomplete product traceability, fragmented care, medical tourism, or procedures performed outside standardized quality systems may further increase diagnostic uncertainty and continuity-of-care problems [6,10].

From an economic perspective, the burden of infection-related complications after minimally invasive aesthetic procedures remains insufficiently characterized, although available studies suggest that infections after cosmetic facial injections or cosmetic tourism may generate substantial socioeconomic and financial consequences [11,12]. Available literature has primarily focused on clinical manifestations, causative microorganisms, diagnostic challenges, and therapeutic strategies, whereas fewer reports have translated these complications into structured pathways of health care resource use or potentially avoidable cost domains [3-7]. This distinction is important because the economic impact of these events may extend beyond the initial infectious episode and may include repeated consultations, delayed microbiological diagnosis, empirical treatment failure, imaging studies, biopsy, drainage, debridement, material removal, prolonged antimicrobial therapy, specialist follow-up, aesthetic sequelae, psychological distress, and loss of continuity of care [12].

Biofilm-associated complications add further clinical and economic complexity in procedures involving dermal fillers or other materials deposited in soft tissues, as persistent nodules, recurrent inflammation, delayed abscesses, and partial response to empirical therapy may be misclassified as noninfectious inflammatory reactions, thereby delaying microbiological evaluation and targeted management [7].

From a clinical-economic perspective, the available literature on infection-related complications after aesthetic procedures supports the need to characterize the clinical pathway, identify resource-use categories, distinguish acute from delayed or material-associated presentations, and recognize that the burden may extend beyond the initial infectious episode to include diagnostic delay, repeated empirical treatment, specialist referral, procedural management, prolonged follow-up, sequelae, and resource-related consequences [3-7]. However, these elements have not been specifically operationalized as an integrated framework for minimally invasive aesthetic procedures, where failures in asepsis, product traceability, early microbiological diagnosis, referral timing, and continuity of care may drive preventable diagnostic and therapeutic escalation [5,6]. The novelty of the present framework lies in adapting this clinical and economic evidence to a procedure-specific patient-safety pathway in aesthetic medicine, linking modifiable care-process failures with infectious outcomes, resource use, and potentially avoidable cost domains.

For these reasons, infection-related complications after minimally invasive aesthetic procedures require an integrated approach that connects patient safety, clinical microbiology, product traceability, infection prevention, diagnostic timeliness, and health care resource use [2,7,10]. This technical report proposes a clinical-economic framework designed to organize the relationship between care-process failures, infectious outcomes, clinical consequences, resource utilization, and potentially avoidable cost domains. The objective is not to perform a formal economic analysis, estimate universal costs, or demonstrate cost-effectiveness, but to provide an operational framework that may support clinical audits, risk management, quality improvement, infection prevention, timely microbiological diagnosis, and future research on the economic consequences of these complications.

## Technical report

Framework development and literature selection

This technical report was developed through a targeted, evidence-informed synthesis of literature on minimally invasive aesthetic procedures, infectious complications, nontuberculous mycobacterial infections, biofilm-associated filler complications, medical tourism-related infections, infection-prevention practices, quality standards in aesthetic medicine, and economic consequences of post-procedural complications. The evidence base included publications on procedural safety and acute bacterial infections after nonsurgical cosmetic procedures, nontuberculous mycobacterial infections and medical tourism-related infections, biofilm-associated delayed filler complications, infection-prevention practices and quality standards in aesthetic or dermatologic procedures, and socioeconomic or financial consequences of cosmetic procedure-related infections. Relevant sources were identified through focused biomedical searches and review of key publications addressing clinical presentation, microbiological diagnosis, resource use, prevention failures, traceability, and continuity-of-care problems in aesthetic or dermatologic procedures.

Sources were selected according to their relevance to framework development, rather than through formal systematic review methodology. Priority was given to clinical guidance and quality standards, systematic reviews, case-based evidence on atypical or delayed infections, infection-prevention studies, and publications describing resource use or financial implications of infectious complications. Because the objective was to develop an operational clinical-economic framework, no meta-analysis, risk-of-bias assessment, or formal economic modeling was performed.

Conceptual basis of the clinical-economic framework

The proposed clinical-economic framework is based on three interdependent domains: clinical burden, health care resource use, and potentially avoidable cost domains. Within this framework, clinical burden is operationally defined as the medical, functional, aesthetic, and psychosocial consequences attributable to an infection-related complication after a minimally invasive aesthetic procedure. These consequences may include acute inflammatory or suppurative manifestations [2], delayed nodular or granulomatous presentations related to atypical microorganisms [3-6], tissue damage or material-associated complications [7], aesthetic sequelae, patient distress, and deterioration of continuity of care [11,12].

Health care resource use refers to the clinical encounters, diagnostic tests, therapeutic interventions, procedures, referrals, and follow-up activities required to evaluate, control, or resolve the complication. This domain includes the resources used during initial assessment, microbiological evaluation, tissue or imaging diagnosis, pharmacological treatment, procedural management, and longitudinal monitoring, which are recurrent elements in the management of acute bacterial infections, nontuberculous mycobacterial infections, biofilm-suspected complications, and medically complex post-procedural infections [2-7].

**Figure 1:**
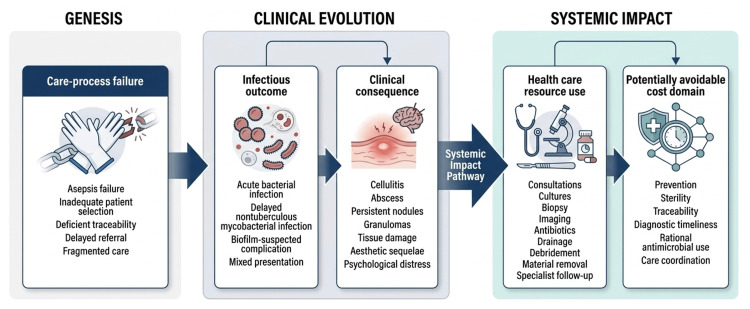
Conceptual Pathway of the Clinical-Economic Framework for Infection-Related Complications After Minimally Invasive Aesthetic Procedures Illustrates the proposed pathway linking modifiable failures in the aesthetic care process with infectious outcomes, clinical consequences, health care resource use, and potentially avoidable cost domains. The model is not intended to assign monetary values or infer demonstrated savings, but to support structured case review, documentation audits, risk management, quality improvement, and prospective data collection. Created using Microsoft PowerPoint and Canva

Potentially avoidable cost domains are defined as categories of resource consumption that may be linked to preventable or modifiable failures in the care process. They should not be interpreted as proven savings, confirmed preventable expenditure, or cost-effectiveness estimates. Instead, they identify areas where better prevention, patient selection, aseptic technique, product traceability, documentation, patient education, timely diagnostic evaluation, rational antimicrobial use, and early referral may reduce unnecessary clinical complexity and resource consumption. These domains are supported by evidence on infection prevention, procedural quality, traceability, fragmented care, medical tourism, and financial consequences of post-cosmetic procedure infections [5,6,8]. The overall conceptual pathway of the proposed clinical-economic framework is shown in Figure 1.

Infectious complication pathways after minimally invasive aesthetic procedures

The framework organizes infection-related complications into operational pathways according to timing, clinical pattern, suspected mechanism, and resource implications. The first pathway corresponds to acute bacterial infection. This pathway is considered when symptoms appear early after the procedure and is characterized by erythema, pain, edema, local warmth, folliculitis, cellulitis, suppuration, or abscess formation. In this pathway, the main operational concern is whether the event reflects a failure in primary prevention, aseptic technique, supply handling, sterilization, or early post-procedure recognition [2,8,9].

The second pathway corresponds to subacute or delayed infection. This pathway is considered when the clinical course is indolent, recurrent, nodular, granulomatous, suppurative, poorly responsive to conventional empirical therapy, or suggestive of atypical microorganisms. The operational priority is to avoid repeated empirical management without appropriate microbiological evaluation, tissue assessment, or timely specialist referral, particularly when rapidly growing nontuberculous mycobacteria are part of the differential diagnosis [3-6].

The third pathway corresponds to material-associated or biofilm-suspected complications. This pathway is considered when symptoms occur in relation to material implanted, injected, or deposited in soft tissues and present as persistent nodules, recurrent inflammation, delayed abscesses, partial therapeutic response, or clinical relapse. In this pathway, the central operational issue is whether the condition has been misclassified as a purely noninfectious inflammatory reaction, delaying appropriate diagnostic and procedural management [7].

These pathways are not mutually exclusive. A patient may present with overlapping features of acute infection, delayed infection, material-associated disease, and noninfectious inflammation. For this reason, the framework emphasizes chronological reconstruction, documentation review, microbiological suspicion, and structured classification before the complication evolves into a prolonged sequence of consultations, empirical treatments, procedures, sequelae, and fragmented care [2-7].

Health care resource use domains

Health care resource use is organized into six operational domains: initial clinical care, microbiological diagnosis, tissue or imaging evaluation, pharmacological treatment, therapeutic procedures, and longitudinal follow-up. These domains reflect recurrent diagnostic, therapeutic, procedural, and follow-up requirements described in acute bacterial soft tissue infections, nontuberculous mycobacterial infections, biofilm-associated filler complications, and financially burdensome post-cosmetic procedure infections [2-5].

Initial clinical care includes first-contact assessment, identification of warning signs, classification of timing and severity, evaluation of the procedure performed, review of product traceability, and determination of whether the presentation is compatible with expected post-procedure inflammation, acute infection, delayed infection, material-associated disease, or mixed presentation. This domain is particularly relevant in aesthetic procedures involving fillers, injectable products, or outpatient interventions where early recognition, documentation, and quality standards influence subsequent management [1,2,10].

Microbiological diagnosis includes the collection and processing of appropriate clinical specimens when infection is suspected, particularly in cases with suppuration, abscess, recurrent lesions, delayed presentation, nodular or granulomatous disease, therapeutic failure, or previous antibiotic exposure. This domain is intended to support targeted management and reduce repeated empirical treatment, especially when common bacterial infection, nontuberculous mycobacterial infection, or postoperative infection associated with cosmetic tourism is suspected [2-6].

Tissue or imaging evaluation includes diagnostic procedures used to characterize depth, extension, collections, residual material, fistulous tracts, granulomatous reaction, or involvement of adjacent structures. This domain is especially relevant when physical examination is insufficient to determine the extent of disease, when atypical infection or biofilm-associated complication is suspected, or when procedural intervention is being considered [3-5].

Pharmacological treatment includes empirical therapy, culture-guided adjustment, combination regimens when required, monitoring for therapeutic response, toxicity surveillance, and treatment modification in case of clinical failure. This domain also includes the evaluation of inappropriate or repeated antimicrobial exposure as a contributor to avoidable resource use, particularly when delayed microbiological diagnosis leads to ineffective empirical treatment or prolonged therapeutic escalation [11,12].

Therapeutic procedures include drainage, incision, debridement, removal of implicated material when clinically indicated, corrective interventions, and other procedural measures required to control infection, reduce tissue damage, or manage sequelae. These interventions are especially relevant in abscesses, atypical mycobacterial infections, material-associated complications, and complex infections following cosmetic procedures or cosmetic tourism [2-7,12].

Longitudinal follow-up includes clinical monitoring, documentation of response, management of relapse or persistence, evaluation of aesthetic sequelae, assessment of patient-reported impact, coordination among specialties, and closure of the adverse-event pathway. This domain is clinically relevant because delayed or atypical infections may require prolonged follow-up, while socioeconomic and financial consequences may persist beyond the initial infectious episode [3,4].

Potentially avoidable cost domains

Potentially avoidable cost domains are grouped according to the point at which a modifiable failure may occur. Primary prevention and biosafety failures include insufficient antisepsis, inadequate hand hygiene, improper personal protective measures, inadequate environmental control, contamination of supplies, inappropriate handling of vials or syringes, and deficient procedural preparation. These domains are supported by evidence on infection risks, professional perceptions of prevention measures, and infection-control practices in aesthetic or dermatologic procedure settings [8,9].

Supply control and sterility failures include reuse or inappropriate handling of materials, contamination of opened products, inadequate sterilization control, or insufficient verification of equipment and supplies. These failures may increase the probability of infection-related events and subsequent resource use, particularly when procedural environments lack standardized quality controls or infection-prevention protocols [8-10].

Documentation and traceability failures include incomplete recording of the product, lot, supplier, expiration date, anatomical site, technique, anatomical plane, administered volume, number of punctures, procedural conditions, and post-procedure instructions. Incomplete traceability increases uncertainty during complication management and may lead to repeated diagnostic testing, additional consultations, or delayed decisions, especially in cases involving injected materials, fragmented care, medical tourism, or products without reliable procedural documentation [1,5,6,10,12].

Pre-procedure assessment failures include performing procedures in patients with active local infection, inflammatory skin disease at the procedure site, relevant immunosuppression, previous procedure-related complications, recent interventions in the same anatomical region, or barriers to adequate follow-up. This domain is aligned with procedural safety principles, prevention of acute bacterial complications, and quality standards in aesthetic medicine and surgery [1,2,10].

Early diagnostic failures include delayed recognition of infection, underestimation of atypical or delayed presentations, interpretation of persistent nodules as exclusively inflammatory, repeated empirical treatment without diagnostic confirmation, delayed microbiological testing, delayed tissue evaluation, and late specialist referral. These failures are particularly relevant in acute bacterial infections, nontuberculous mycobacterial infections, and biofilm-associated complications, where delayed diagnosis may increase therapeutic complexity and resource use [2-7].

Continuity-of-care failures include fragmented follow-up, absence of a defined pathway for complications, incomplete transfer of procedural information, lack of access to the original provider, medical tourism-related discontinuity, unregulated procedures, and products without reliable documentation. These failures are clinically and economically important because they may increase diagnostic uncertainty, duplicate resource use, delay definitive management, and contribute to financial burden after cosmetic procedure-related infections [5,6].

Patient education and follow-up failures include insufficient communication of warning signs, unclear post-procedure instructions, delayed consultation, self-medication, poor adherence to follow-up, and failure to recognize progression of symptoms. This domain is relevant to prevention, early recognition, timely referral, and quality improvement in nonsurgical cosmetic and aesthetic procedures [2,10].

Proposed clinical-economic framework

The proposed framework organizes five sequential components: process failure, infectious outcome, clinical consequence, health care resource use, and potentially avoidable cost domain. It is intended to function as a structured clinical and managerial tool for case review, prospective risk assessment, documentation audits, quality-improvement activities, and institutional learning. The framework does not assign monetary values, estimate direct or indirect costs, or infer demonstrated cost savings. Instead, it identifies points in the care process where preventable or modifiable failures may generate additional clinical burden, diagnostic complexity, therapeutic escalation, and resource consumption. The complete clinical-economic framework and its proposed operational domains are summarized in Table 1.

**Table 1:** Clinical-Economic Framework for Potentially Avoidable Infection-Related Costs After Minimally Invasive Aesthetic Procedures

Process failure	Infectious outcome	Clinical consequence	Health care resource use	Potentially avoidable cost domain
Deficient antisepsis or hand hygiene	Acute bacterial infection	Cellulitis, folliculitis, abscess, local suppuration	Consultation, culture, antibiotics, drainage, follow-up	Primary prevention and biosafety
Reuse, inappropriate handling, or contamination of supplies	Inoculation of skin flora or environmental microorganisms	Abscess, soft tissue infection, possible outbreak	Emergency care, cultures, antibiotics, local intervention, epidemiological surveillance	Supply control and sterility
Nonvalidated sterilization or insufficient autoclave control	Transmission of environmental or resistant bacteria	Post-procedure infection, abscesses, recurrence	Culture, antimicrobial susceptibility testing, antibiotics, debridement	Sterilization validation
Incomplete documentation of product, lot, supplier, or technique	Causal uncertainty after infection	Difficulty deciding on material removal or specific management	Repeat consultations, imaging, specialist referrals, additional documentation	Documentation and traceability
Inadequate patient selection	Increased susceptibility to infection or mixed complications	Local progression, poor response, greater clinical complexity	Additional consultations, complementary studies, close follow-up	Pre-procedure assessment
Low suspicion of nontuberculous mycobacteria	Subacute or delayed infection	Persistent nodules, cold abscesses, granulomas, failure of common antibiotics	Mycobacterial culture, biopsy, susceptibility testing, prolonged combination therapy	Timely microbiological diagnosis
Interpretation of persistent nodules as noninfectious inflammation	Unrecognized biofilm or material-associated infection	Recurrent nodules, granulomas, delayed abscesses	Ultrasound, biopsy, hyaluronidase, antibiotics, surgery	Early differential diagnosis
Repeated empirical management without culture or referral	Persistent or progressive infection	Relapse, chronic inflammation, greater tissue damage	New consultations, additional antibiotics, complementary studies	Rational antimicrobial use
Insufficient post-procedure follow-up or patient education	Delayed consultation for warning signs	Progression of cellulitis, abscess, or atypical infection	Emergency care, therapeutic procedures, serial follow-up visits	Patient education and follow-up
Medical tourism, fragmented care, or products without traceability	Infection with incomplete technical information	Unknown product, lack of continuity, management under uncertainty	Repeated diagnostic testing, imaging, cultures, multiple specialists	Care coordination and regulation

The framework should be applied through a stepwise reconstruction of the event. First, the procedure should be characterized according to the product or material used, anatomical site, technique, timing, aseptic conditions, traceability records, and post-procedure instructions. Second, the clinical course should be organized chronologically, including symptom onset, lesion morphology, warning signs, empirical treatments, microbiological tests, imaging studies, procedures, referrals, and follow-up. Third, the complication should be assigned to the most plausible operational pathway: acute bacterial infection, subacute or delayed infection, material-associated or biofilm-suspected complication, noninfectious inflammatory reaction, or mixed presentation. This approach is consistent with the need to integrate procedural safety, microbiological suspicion, documentation, traceability, and continuity of care when evaluating infectious complications after aesthetic procedures [5-7,10,12].

Once the pathway is defined, resource use can be mapped across initial clinical care, microbiological diagnosis, tissue or imaging evaluation, pharmacological treatment, therapeutic procedures, and longitudinal follow-up. This mapping allows the care team to distinguish resources primarily required by the clinical severity of the complication from resources potentially driven by delayed recognition, incomplete documentation, fragmented care, inadequate traceability, or repeated empirical management [2,11,12].

The potentially avoidable cost domain should be interpreted as a risk-management category, not as evidence that expenditure was preventable in every individual case. The framework therefore supports identification of modifiable points in the process, including prevention, aseptic technique, supply handling, sterilization validation, product traceability, patient selection, patient education, timely microbiological evaluation, rational antimicrobial use, and early specialist referral. These points correspond to prevention and quality failures [8-10], diagnostic and therapeutic escalation in infectious complications, and resource or financial consequences described after cosmetic procedure-related infections.

Prospectively, the framework may be incorporated into pre-procedure checklists, informed consent processes, traceability forms, post-procedure warning instructions, complication pathways, pharmacovigilance or technovigilance records, infection-control audits, and institutional quality-improvement programs. Retrospectively, it may support root-cause analysis, classification of adverse events, evaluation of documentation completeness, and identification of recurrent failures across cases. These applications are aligned with infection-prevention practices, quality standards, documentation needs, and the need to characterize resource use in post-procedural infectious complications.

The main contribution of the framework is to convert infection-related complications after minimally invasive aesthetic procedures into structured, auditable events. By linking process failures with clinical consequences and resource-use domains, the model provides a practical basis for prevention, early recognition, coordinated management, institutional learning, and future data collection on costs and outcomes.

## Discussion

Infections following minimally invasive aesthetic procedures are commonly described from microbiological, dermatological, or surgical perspectives, particularly in relation to acute bacterial soft tissue infections, nontuberculous mycobacterial infections, biofilm-associated filler complications, and postoperative infections associated with cosmetic procedures or medical tourism [2,3]. Although these approaches are clinically necessary, they may underestimate the broader impact of these complications when the full sequence of diagnostic, therapeutic, procedural, follow-up, sequelae-related, and resource-use consequences is not considered [11,12]. The proposed framework addresses this gap by linking care-process failures with infectious outcomes, clinical consequences, health care resource use, and potentially avoidable cost domains.

This approach is relevant because infection-related complications after aesthetic procedures do not follow a single clinical pathway. Acute bacterial infections may present as cellulitis, abscesses, folliculitis, erythema, pain, edema, or local suppuration, usually after disruption of the skin barrier and inoculation of skin microbiota or environmental microorganisms [2]. In contrast, rapidly growing nontuberculous mycobacterial infections may present with delayed, indolent, nodular, granulomatous, suppurative, or antibiotic-refractory lesions. Low initial suspicion for these pathogens may result in repeated consultations, empirical antimicrobial exposure, delayed cultures, late biopsy, and prolonged combined antimicrobial therapy [3-5].

Biofilm-associated infection adds further complexity, particularly in procedures involving dermal fillers or other materials deposited in soft tissues [1,7]. These complications may mimic noninfectious inflammatory reactions, delayed hypersensitivity, or sterile granulomas, which can delay microbiological evaluation, imaging, biopsy, procedural intervention, material removal when clinically indicated, and targeted treatment [7]. From an economic perspective, the relevant burden is therefore not limited to the cost of antibiotics, consultations, or isolated procedures. It may include the cumulative cascade of delayed recognition, incomplete diagnosis, fragmented care, prolonged treatment, repeated interventions, aesthetic sequelae, and loss of continuity of care [11,12].

The framework is also relevant because several drivers of resource use are potentially modifiable. In aesthetic medicine, procedures may occur in outpatient environments with heterogeneous levels of professional training, infection-prevention protocols, product traceability, documentation standards, sterilization control, and post-procedure follow-up. Previous evidence has identified variability in biosafety practices, environmental control, hand hygiene, and written prevention protocols in settings where aesthetic or dermatologic procedures are performed [8]. These factors support the need to analyze infectious complications not only as isolated biological events, but also as safety and quality-of-care events [8,9].

Documentation and traceability are particularly important for clinical decision-making. Absence of information on the product used, lot number, supplier, expiration date, anatomical site, injected volume, anatomical plane, number of punctures, procedural technique, aseptic conditions, and post-procedure instructions may increase diagnostic uncertainty and delay appropriate management [1,10]. This problem becomes more pronounced in contexts of medical tourism, fragmented care, unregulated providers, or products without reliable traceability, where the clinician managing the complication may not have access to essential procedural information [5,6,12].

From a patient safety perspective, the proposed framework may be applied both prospectively and retrospectively. Before the procedure, it can support verification of patient selection, skin condition, absence of active local infection, aseptic technique, sterilization control, product traceability, informed consent, and post-procedure warning instructions [1,2,8]. After the procedure, it may help classify complications according to timing, clinical pattern, suspected mechanism, and resource implications, supporting decisions regarding microbiological testing, biopsy, imaging, specialist referral, procedural management, and longitudinal follow-up [2-7].

This report has limitations. It does not constitute a formal economic evaluation, does not estimate direct or indirect costs in a specific currency, and does not demonstrate cost savings. It also does not quantify the incidence of infectious complications after minimally invasive aesthetic procedures, because the available evidence is heterogeneous, procedure-specific, and likely affected by underreporting. Therefore, the term “potentially avoidable cost” should be interpreted as an operational risk-management category rather than as proof that all associated expenditures are preventable.

Despite these limitations, the framework offers a pragmatic structure for organizing a clinical problem that intersects aesthetic medicine, dermatology, plastic surgery, infectious diseases, microbiology, patient safety, and health care management. Future studies should develop prospective registries that capture procedure type, product characteristics, traceability data, technique, microbiological findings, diagnostic tests, therapeutic interventions, episode duration, complications, sequelae, patient-reported impact, and resource use. Such data would allow progression from a conceptual framework toward more robust, comparable, and context-specific economic models. Prospective validation is required before the framework can be recommended for widespread implementation, including assessment of feasibility, interobserver consistency, completeness of documentation, ability to classify complications, and usefulness for identifying modifiable drivers of resource consumption.

## Conclusions

Infections following minimally invasive aesthetic procedures may generate a disproportionate clinical and economic burden when they are diagnosed late, treated with repeated empirical regimens, or managed in fragmented care settings. The proposed clinical-economic framework allows these complications to be analyzed as auditable events by linking care-process failures with infectious outcomes, clinical consequences, health care resource use, and potentially avoidable cost domains.

Infection prevention, product traceability, aseptic technique, patient education, timely microbiological diagnosis, and early specialist referral should be considered central components of safety in aesthetic medicine. This technical report does not replace a formal economic analysis, but it offers an applicable structure to identify critical intervention points, reduce diagnostic delays, improve quality of care, and guide future research on costs, outcomes, and prevention of infectious complications after minimally invasive aesthetic procedures. Prospective validation studies are needed to determine the feasibility, reproducibility, and clinical utility of the framework before broader implementation in routine aesthetic medicine practice.
